# Women’s autonomy in refusing risky sex and associated factors in Ethiopia: evidence from 2011 to 2016 EDHS data

**DOI:** 10.1186/s12905-021-01479-y

**Published:** 2021-09-16

**Authors:** Melkamu Dires Asabu

**Affiliations:** Department of Political Science and International Relations, Faculty of Social Sciences and Humanities, Woldia University, P.O.Box 400, Woldia, Ethiopia

**Keywords:** Autonomy, Ethiopia, Refusing, Risky sex, Women

## Abstract

**Background:**

Risky sexual behavior is a major public health concern of Ethiopians. Although studying the autonomy of women in refusing risky sex is significant to take proper actions, the issue is not yet studied. Accordingly, this population-based nationwide study was aimed at assessing women’s autonomy in refusing risky sex and its associated factors in Ethiopia.

**Method:**

The sample was limited to married women of 2011 (n = 8369) and 2016 (n = 8403) Ethiopian Demographic and Health Survey data. Women's autonomy in refusing risky sex was measured based on wives' response to 'not having sex because husbands have other women. To examine associated factors, socio-demographic variables were computed using binary logistic regression.

**Result:**

The finding revealed that the trend of women’s autonomy in refusing risky sex had declined from 78.9% in 2011 to 69.5% in 2016. Women aged from 25 to 34 were less likely autonomous in refusing sex compared to those who aged less than 24 years old (AOR = .7064; 95% CI 0.605, 0.965). The autonomy of women with higher educational status was three times more likely higher than those who have no formal education (AOR = 3.221; 95% CI 1.647, 6.300 respectively. The autonomy of women who are from rich households was more likely higher in comparison to women from poor households (AOR = 1.523; 95% CI 1.28, 1.813). The autonomy of women those who live in Tigray 2.9 times (AOR = 2.938; 95% CI 2.025, 4.263), Amhara 4.8 times (AOR = 4.870; 95% CI 3.388, 7.000), SNNP 1.9 times (AOR = 1.900; 95% CI 1.355, 2.664), and Addis Ababa 3.8 times (AOR = 3.809; 95% CI 2.227, 6.516) more likely higher than those who reside in Dire Dawa.

**Conclusion:**

The autonomy of women in refusing risky sex has declined from 2011 to 2016. This infers that currently, women are more victimized than previously. Hence, possible interventions like empowering women shall be taken to protect women from certain health problems of risky sexual behavior.

## Introduction

Risky sexual behavior is defined as an individual’s practice in one or more of the following acts such as unprotected sexual intercourse, starting sexual activity at a young age, having multiple sexual partners, having sex under the influence of stimulant substances, or having sex immediately after watching pornographic [1–4]. Risky sexual behavior is a major public health concern across the world but the issue is more serious in developing countries including Ethiopia. It increases the likelihood of individuals’ vulnerability in sexually transmitted infections (STIs), HIV/AIDS, unwanted pregnancy, and psychological distress [2, 5–8].

Having multiple sexual partners refers that an individual’s sexual interaction with two or more sexual partners that overlapped in time [9–11]. Since having multiple sexual partners is risky sexual behavior and a key driver of HIV/AIDS transmission, those who have concurrent sexual partners increase their risk of contracting HIV/AIDS. Moreover, actors of multiple sexual partners are more likely exposed to sexually transmitted infections (STIs). Thus, since having multiple sexual partners is risky sex, measures not to have sexual intercourse with those who have multiple sexual partners can be understood as a refusal of risky sex.

Autonomy can be defined as a technical, social and psychological ability of an individual for making decisions about his/her private concerns [12]. An individual is autonomous when she/he can act under her/his direction, i.e. make her/his actions [13]. Autonomous women can refuse risky sex if husbands have risky sexual behavior like having sexual contact with an additional woman. This is to protect themselves from adverse effects of risky sexual behavior like sexually transmitted infections (STIs) and HIV/AIDS. Concerning women’s autonomy, research and policy discourse indicated the presence of limited autonomy of women in developing countries and also the challenge of their lower autonomy to improve their reproductive health [14, 15].

Human rights instruments underscore that wives have the right to refuse sexual intercourse with their husbands not only if they are not interested to do that but also when husbands do have a sexual relationship with additional women. Refusing risky sex is a means to enable women to exercise their right to human dignity, right to health, right to bodily integrity as well as the right not to be subjected to cruel, inhuman, and degrading treatment [16, 17]. In a study conducted in Ethiopia among Preparatory School Students, the magnitude of risky sexual behaviour particularly in having multiple sexual partners estimated up to 53.7% [18]. To address the problem increasing the autonomy of women in refusing risky sex is very crucial. This is due to the fact that women’s bargaining power determines their decision-making status [19–21].

In Ethiopia, different activities might be done to empower women; however, as far as the researcher’s knowledge is concerned, little attention has given for women’s autonomy in refusing risky sex. This is might be due to the fact that the level of women’s autonomy in refusing risky sex and its associated factors is not well studied in the country. Understanding women’s autonomy in refusing risky sex and its associated factors is necessary at least because of the following two reasons. These are firstly, to take possible interventions like empowering women; and secondly, to minimize the risk of sexually transmitted diseases due to multiple sexual partners. Accordingly, this study objective was focused, using the 2011 and 2016 Ethiopian DHS data, on examining women’s autonomy in refusing risky sex and associated factors in Ethiopia. The finding will help to understand women’s autonomy from time to time in one hand and it is also important to take proper intervention for women’s health on the other hand.

## Objectives of the study

Using 2011 & 2016 EDHS data, the study’s objectives were: (1) to examine women’s autonomy in refusing risky sex in Ethiopia; and (2) to identify associated factors with women’s autonomy in refusing risky sex in Ethiopia.

## Methods

### Study design and data collection

It is a population-based cross-sectional study design based on the 2011 and 2016 EDHS data. The study used data from the 2011 and 2016 EDHS that were collected by the Central Statistical Authority (CSA) of Ethiopia and Opinion Research Corporation Company (ORC) Macro International. It was conducted in all Regional States of Ethiopia namely Tigray, Afar, Amhara, Oromia, Somali, Benishangul Gumuz, Southern Nations Nationalities and Peoples (SNNP), Gambella and Harari, and in Addis Ababa, and Dire Dawa city Administrations (CSA and ICF, 2016) [22]. It is a nationally representative sample survey, aged 15–49 years’ women.

By considering its national representativeness, the sampling method for the 2016 EDHS sample was stratified and selected in two stages [23]. Each region was stratified into urban and rural areas, yielding 21 sampling strata. The survey collected a detailed woman's background characteristics. The survey also collected information from unmarried, married, living with a partner, divorced, and widowed women. However, for this study, the researcher has used only married women’s data. In this study, spouses from all types of marital unions (religious, cultural, and municipality) were included. Based on the valid number of responses for identified variables, the sample size of the study from 2011 to 2016 DHS data was limited to 8369 and 8403 respectively.

### Variables and measurement

#### Dependent variable

The study’s dependent variable was women's autonomy in refusing risky sex. This was measured based on women’s response to ‘Reason for not having sex because of husbands have other women’ [24, 25]. Wives who can refuse sex if husbands have other women were considered as ‘autonomous in refusing risky sex’ and wives who cannot refuse sex if husbands have other women were considered as ‘not autonomous in refusing risky sex’. Finally, the dependent variable that dichotomized as ‘‘autonomous in refusing risky sex’, and ‘not autonomous in refusing risky sex’ was coded as “0” and “1” respectively.

#### Independent variables

The study identified the following independent variables including women’s age, education status, working status, place of residence, household wealth index, religion, and region. The researcher adopted the measurements of the DHS survey for the following four independent variables. However, the measurements of the DHS survey on the following variables including age, education level, and household wealth index were adapted as follows.

The adapted measurements include (1) age of respondents that was open to writing their exact age, but the study that focused on modern contraceptive use measured age of label age of respondents by labeling from aged 15–24, 25–34, and 35–49 [26]. Since there are few women in marriage since the age of 11, this study used 11–24, 25–34, and 35–49 age categories of women. (2) For educational attainment, the DHS used six responses such as no education, incomplete primary, primary, incomplete secondary, secondary, and higher. As studies were done using DHS data on "the effect of maternal health service utilization in early initiation of breastfeeding among Nepalese mothers" [27]. as well as “women empowerment and their reproductive behavior among currently married women in Ethiopia” [4] have used 'illiterate', 'primary', 'secondary' and 'higher' to measure this variable, for this study, incomplete primary and primary, and incomplete secondary and secondary merged into 'primary' and 'secondary' respectively. (3) Concerning the wealth index, the middle was taken as it is but the categories poorest and poor, and rich and richest were merged into poor and rich respectively. Similarly, other studies [28–31] have used these variables to measure the wealth index.

### Method of data analysis

The data obtained from 2011 to 2016 EDHS were analyzed through SPSS version 22 in three levels. First, the univariate/descriptive statistics were used to summarize the socio-demographic variables of the study participants using frequency and percentages. Second, the bivariate analysis was done using the chi-square test (*p* < 0.05) to identify the socio-demographic variables that were significantly associated with women's autonomy in refusing risky sex. Finally, analysis of the determinants of women's autonomy in refusing risky sex was carried out using logistic regression. This is because logistic regression is used to examine the relationships between a categorical outcome variable and one or more categorical or continuous predictor variables [32]. Principally, binary logistic regression is applied in cases where the dependent variable is dichotomous [33]. This is because the dependent variable (women autonomy in refusing risky sex) was dichotomized as “not autonomous in refusing risky sex” and “autonomous in refusing risky sex”.

For binary logistic regression analyses, statistical inferences were made based on estimates of the odds ratio (OR) with a 95% confidence level and 5% margin of error or p-value less than 0.05. The study used an unadjusted odds ratio to estimate the gross effect of each independent variable on the outcome variable. The independent variables that had an association of a p-value less than 0.05 with the outcome variable were taken for the multiple or adjusted analysis.

Before reporting the result of the adjusted odds ratio, the overall goodness of fit was assessed via the Hosmer–Lemeshow test. The result of this analysis's *P* value (0.606) was greater than the level of significance α = 0.05, hence the data fit the model well. Because in the Hosmer–Lemeshow test, an insignificant chi-square indicates a good fit to the data [34]. In addition, the final model of the logistic regression was assessed for its robustness using methods of checking multicollinearity. It could be checked using the following three methods such as correlation matrices, tolerance, and variance of inflation factors (VIF) [35]. There is a problem of multicollinearity if the correlation of any two variables is 0.8 and more [35, 36]. The result of the correlation matrix analysis of this study was less than 0.8, which indicates an absence of the problem of multicollinearity. In this study, therefore, these indict an absence of multicollinearity problem [35, 37]. The study’s tolerance and VIF values were more than 0.1 and less than 10 respectively. These also indicate an absence of a problem of multicollinearity.

## Results

**Fig. 1:**
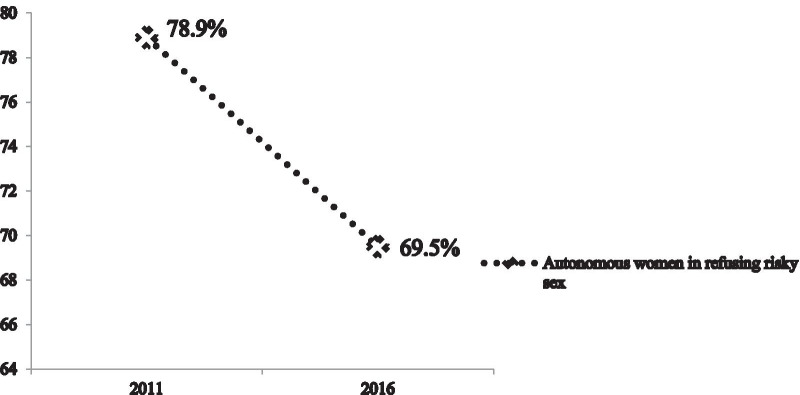
Women’s autonomy in refusing risky sex

As we have seen in Fig. 1, the autonomy of women in refusing risky sex has declined from 78.9% in 2011 to 69.5% in 2016.

As it has shown in Table 1, the percentage of autonomous women refusing risky sex in 2011 was 78.9%, while in 2016 it has declined to 69.5%. There is a significant decrement of women's autonomy in refusing risky sex. When the age of women and their autonomy in refusing risky sex directly associated in 2011 but there is up and down in 2016. Women's educational status and their autonomy in refusing risky sex were directly associated in both 2011 and 2016. In both 2011 and 2016, the autonomy of employed women in refusing risky sex was better than housewife/unemployed women albeit its percentage is not identical. Regarding residence, the percentage of rural women autonomous in refusing risky sex was 89.6% and 79.8% in 2011 and 2016 were respectively. Similar to the educational status of women, in both 2011 and 2016, the numbers of autonomous women refusing risky sex increased from poor to rich wealth index.

**Table 1:** Description of socio-demographic variables of married women

Background characteristics of women	Women autonomy in refusing risky sex			
	2011 n = 8369		2016 n = 8403	
	Not autonomous	Autonomous	Not autonomous	Autonomous
*Age*				
< 24	1646 (21.3%)	6085 (78.7%)	2370 (30.7%	5356 (69.3%)
25–34	121 (19.5%)	499 (80.5%)	190 (28.7%)	473 (71.3%)
35–49	3 (16.7%)	15 (83.3%)	5 (35.7%)	9 (64.3%)
*Educational status*				
No education	1405 (24.7%)	4275 (75.3%)	1857 (36.2%)	3269 (63.8%)
Primary	330 (16.0%)	1732 (84.0%)	537 (24.4%)	1667 (75.6%)
Secondary	28 (7.2%)	359 (92.8%)	123 (18.1%)	555 (81.9%)
Higher	7 (2.9%)	233 (97.1%)	48 (12.2%)	347 (87.8%)
*Working status*				
Housewife	1321 (23.2%)	4361 (76.8%)	1805 (31.7%)	3892 (68.3%)
Employed	449 (16.7%)	2238 (83.3%)	760 (28.1%)	1946 (71.9%)
*Residence*				
Urban	189 (10.4%)	1637 (89.6%)	399 (20.2%)	1577 (79.8%)
Rural	1581 (24.2%)	4962 (75.8%)	2166 (33.7%)	4261 (66.3%)
*Wealth Index*				
Poor	1046 (28.2%)	2659 (71.8%)	1465 (38.5%)	2338 (61.5%)
Middle	265 (19.9%)	1068 (80.1%)	360 (29.8%)	847 (70.2%)
Rich	459 (13.8%0	2872 (86.2%)	740 (21.8%)	2653 (78.2%)
*Religion*				
Orthodox	366 (12.2%)	2643 (87.8%)	529 (17.7%)	2454 (82.3%)
Catholic	29 (33.3%)	58 (66.7%)	15 (30.6%)	34 (69.4%)
Protestant	387 (26.2%)	1089 (73.8%)	466 (29.3%)	1126 (70.7%)
Muslim	937 (25.6%)	2728 (74.4%)	1519 (41.5%)	2145 (58.5%)
Traditional	22 (38.6%)	35 (61.4%)	23 (35.4%)	42 (64.6%)
Others	29 (38.7%)	46 (61.3%)	13 (26.0%)	37 (74.0%)
*Region*				
Tigray	93 (10.8%)	766 (89.2%)	120 (14.4%)	711 (85.6%)
Afar	303 (38.7%)	480 (61.3%)	244 (34.8%)	458 (65.2%)
Amhara	131 (11.7%)	984 (88.3%)	107 (10.9%)	879 (89.1%)
Oromia	220 (17.9%)	1011 (82.1%)	490 (41.6%)	688 (58.4%)
Somali	176 (31.8%)	378 (68.2%)	385 (49.5%)	392 (50.5%)
Benishangul Gumuz	201 (26.1%)	568 (73.9%)	353 (49.5%)	360 (50.5%)
SNNP	264 (25.6%)	769 (74.4%)	255 (22.7%)	869 (77.3%)
Gambela	217 (38.1%)	353 (61.9%)	184 (31.0%)	409 (69.0%)
Harari	83 (15.9%)	438 (84.1%)	224 (46.3%)	260 (53.7%)
Addis Ababa	22 (4.7%)	447 (95.3%)	34 (6.4%)	497 (93.6%)
Dire Dawa	60 (12.9%)	405 (87.1%)	169 (34.9%)	315 (65.1%)

In terms of religion, in 2011 the top three autonomous women in refusing risky sex were Orthodox Christian (87.8%), Muslim (74.4%), and Protestant Christian (73.8%) women, but the top three autonomous women in refusing risky sex in 2016 were Orthodox Christian women (82.3%), women from others religion (74.0%) and Protestant Christian women (70.7%).

Less performed women in refusing risky sex were women from other religions (61.3%) and women from the traditional religion (64.6%) in 2011 and 2016 respectively. In the case of geographic areas or regions, the top three autonomous women in refusing risky sex were found in Addis Ababa (95.3%), Tigray (89.2%), and Amhara (88.3%) in 2011. In 2016, the top three autonomous women refusing risky sex were found in Addis Ababa (93.6%), Amhara (89.1%), and Tigray (85.6%). Fewer performance women autonomy in refusing risky sex was recorded from Gambela (61.9%) and Harari (53.7%) regional states’ women in 2011 and 2016 respectively.

As it has shown in Table 2, the study has shown the effect of each variable on the women's autonomy in refusing risky sex in 2016. Among independent variables, two variables such as working status and residence had an insignificant association with women's autonomy in refusing risky sex.

**Table 2:** Logistic regression analysis of associated factors on women autonomy in refusing risky sex (adjusted odds ratio)

Associated factors	Women autonomy in refusing risky sex in 2016	
	COR (95% CI)	AOR (95% CI)
*Age*		
15–24®	1	1
25–34	1.102 (0.925, 1.312)	0.764 (0.605, 0.965)*
35–49	0.796 (0.267, 2.379)	1.213 (0.218, 6.757)
*Education status*		
Illiterate®	1	1
Primary	1.763 (1.575, 1.974)***	1.607 (1.379, 1.874)***
Secondary	2.563 (2.091, 3.142)***	2.208 (1.639, 2.975)***
Higher	4.107 (3.021, 5.583)***	3.221 (1.647, 6.300)**
*Working status*		
Housewife ®	1	1
Employed	1.187 (1.074, 1.313)**	1.463 (0.858, 2.497)
*Residence*		
Urban®	1	1
Rural	0.498 (0.441, 0.562)***	0.950 (0.758, 1.190)
*Wealth Index*		
Poor ®	1	1
Middle	1.474 (1.282, 1.695)***	1.173 (0.975, 1.412)
Rich	2.246 (2.024, 2.494)***	1.523 (1.280, 1.813)***
*Religion*		
Orthodox®	1	1
Catholic	0.489 (0.264, 0.904)*	1.172 (0.516, 2.659)
Protestant	0.521 (0.451, 0.601)***	1.044 (0.816, 1.336)
Muslim	0.304 (0.271, 0.341)***	0.799 (0.652, 0.979)*
Traditional	0.394 (0.235, 0.660)***	1.875 (0.877, 4.009)
Others	0.614 (0.324, 1.162)	1.087 (0.501, 2.359)
*Region*		
Tigray	3.179 (2.429, 4.160)***	2.938 (2.025, 4.263)***
Afar	1.007 (0.790, 1.284)	1.497 (1.111, 2.017)**
Amhara	4.407 (3.350, 5.798)***	4.870 (3.388, 7.000)***
Oromia	0.753 (0.605, 0.939)*	0.845 (0.633, 1.129)
Somali	0.546 (0.432, 0.690)***	0.801 (0.601, 1.068)
Benishangul	0.547 (0.431, 0.694)***	0.568 (0.406, 0.796)**
SNNP	1.828 (1.448, 2.309)***	1.900 (1.355, 2.664)***
Gambela	1.193 (0.924, 1.539)	0.972 (0.669, 1.410)
Harari	0.623 (0.481, 0.806)***	0.516 (0.372, 0.716)***
Addis Ababa	7.842 (5.286, 11.636)***	3.809 (2.227, 6.516)***
Dire Dawa®	1	1

® = Reference category; OR = odds ratio; CI = confidence interval; *p < 0.05; **p < 0.01; ***p < 0.001

However, women aged from 25 to 34 were less likely autonomous in refusing risky sex (AOR = 0.764; 95% CI 0.605, 0.965), in comparison with women aged less than 24 years old. In comparison to illiterate women, the autonomy of women with primary, secondary, and higher educational status were AOR = 1.607; 95% CI 1.379, 1.874, AOR = 2.208; 95% CI 1.639, 2.975, and AOR = 3.221;95% CI 1.647, 6.300. The autonomy of women from rich households was 1.523 times more likely higher in comparison to women from poor households (AOR = 1.523; 95% CI 1.28, 1.813). In the case of religion, when it was compared to Orthodox Christian women's autonomy except for Muslim women's autonomy (AOR = 0.799; 95% CI 0.652, 0.979), the autonomy of women from other stated religions was insignificant. Based on regions, the autonomy of women in Tigray, (AOR = 2.938; 95% CI 2.025, 4.263), Afar (AOR = 1.497; 95% CI 1.111, 2.017), Amhara (AOR = 4.870; 95% CI 3.388, 7.000), Benishangul Gumuz (AOR = 0.568; 95% CI 0.406, 0.796), SNNP (AOR = 1.900; 95% CI 1.355, 2.664), Harari (AOR = 0.516; 95% CI 0.372, 0.716), and Addis Ababa (AOR = 3.809; 95% CI 2.227, 6.516) when compared with an autonomy of women who resides in Dire Dawa.

## Discussion

From 2011 to 2016, the autonomy of women in refusing risky sex has declined from 78.9 to 69.5% (by 9.4%). For this declination of the autonomy of women in refusing risky sex the possible justification might be because of the role of civil society organizations that work in advocating the rights of women had become closed [38].

In comparison with women aged less than 24 years old, older women (25–34 years old) were less likely autonomous in refusing risky sex. Similar to this study finding, on women autonomy in health care decision making [28, 39], household decision making [40], and on controlling and exercising their reproductive rights [41] age had a significant association. The justification might be “older age is associated with decreases in self-esteem” [42].

The finding of this study disclosed the presence of a direct relationship between women's educational status and their autonomy in refusing risky sex. Other studies [15, 43] also found a positive association between women's autonomy with their educational status. This might be due to the fact that ‘highly educated women are more likely to be knowledgeable about their own rights and health, have more self-confidence, and be more assertive than those with less or no education’ [39].

Women's autonomy in refusing risky sex is positively associated with wealth index status. Correspondingly, other studies focused on household decision-making [40, 44] also found a similar result. The probable justification might be when women’s economic status has become improved; their level of self-confidence is also increased. Therefore, since they are not dependent on their husbands, wives can refuse risky sex. Similar to other studies [45, 46] findings, this study also revealed the presence of Muslim women less autonomously. This might be because of " Islam restricts women's freedom to a greater extent than other religions" [47].

## Conclusion and recommendation

The autonomy of women in refusing risky sex has declined from 2011 to 2016. This infers that currently women are more victimized refusing risky sex than previously. Hence, possible interventions like empowering women shall be taken to save women from certain health problems of risky sexual behavior. The study finding revealed that women aged from 25 to 34, illiterate women, women from poor households, Muslim women, women from Benishangul Gumuz and Harari regions were less autonomous in refusing risky sex. Therefore, although declining autonomy of women in refusing risky sex is a serious problem in general, women aged from 25 to 34, illiterate women, women from poor households, Muslim women, women from Benishangul Gumuz and Harari regions in particular needs special attention of stakeholders such as non-governmental organizations, women and children affairs office, and women's association. The roles of these stakeholders might focus on holding open discussions among women and also providing training that can boost women's self-confidence as well as their understanding of women's rights and the impacts of risky sex are categorized under preventive technique. As a curative technique, arranging and providing legal support and guidance and counseling techniques are also essential roles to treat victim women.

## Data Availability

The survey datasets used in this study were based on a publicly available dataset that is freely available at http://dhsprogram.com/data/dataset/Ethiopia_Standard-DHS_2016.cfm?flag=0. The released data is available without participants' identities. Approval was sought from MEASURE DHS/ICF International and permission was granted for this use.
